# Tracing the origin of functional and conserved domains in the human proteome: implications for protein evolution at the modular level

**DOI:** 10.1186/1471-2148-6-91

**Published:** 2006-11-07

**Authors:** Lipika R Pal, Chittibabu Guda

**Affiliations:** 1 Gen*NY*sis Center for Excellence in Cancer Genomics and Department of Epidemiology & Biostatistics, University at Albany, State University of New York, One Discovery drive, Rensselaer, NY 12144–3456, USA

## Abstract

**Background:**

The functional repertoire of the human proteome is an incremental collection of functions accomplished by protein domains evolved along the *Homo sapiens *lineage. Therefore, knowledge on the origin of these functionalities provides a better understanding of the domain and protein evolution in human. The lack of proper comprehension about such origin has impelled us to study the evolutionary origin of human proteome in a unique way as detailed in this study.

**Results:**

This study reports a unique approach for understanding the evolution of human proteome by tracing the origin of its constituting domains hierarchically, along the *Homo sapiens *lineage. The uniqueness of this method lies in subtractive searching of functional and conserved domains in the human proteome resulting in higher efficiency of detecting their origins. From these analyses the nature of protein evolution and trends in domain evolution can be observed in the context of the entire human proteome data. The method adopted here also helps delineate the degree of divergence of functional families occurred during the course of evolution.

**Conclusion:**

This approach to trace the evolutionary origin of functional domains in the human proteome facilitates better understanding of their functional versatility as well as provides insights into the functionality of hypothetical proteins present in the human proteome. This work elucidates the origin of functional and conserved domains in human proteins, their distribution along the *Homo sapiens *lineage, occurrence frequency of different domain combinations and proteome-wide patterns of their distribution, providing insights into the evolutionary solution to the increased complexity of the human proteome.

## Background

One of the biggest challenges in the post-genomic era is to better understand the evolutionary origins of the human proteome. This task can be better accomplished by analyzing the building blocks of proteins (protein domains) rather than entire proteins [1]. Proteins are generally modular in nature where each module or domain is a general designation for recurrent protein fragments with distinct structure, function and/or evolutionary history with autonomous folding and function retaining capability [2,3]. Hence, different proteins can be found with same domain content but with different architectures, or with entirely different domain structures in combination with other domains. It has been suggested that domain combinations are evolutionarily conserved and evolution creates novel functions predominantly by combining existing domains [4,5], often creating Rosetta Stone proteins in different organisms [6]. Moreover, these domain combination networks are known to exhibit small-world and scale-free topologies, where a few domain superfamilies are connected to many different domains, while most domains are adjacent to only one or two types of neighbors [7,8]. Diversity of domain combinations and evolution of domain superfamilies has been attributed to evolutionary processes such as gene recombination, gene duplication, gene fusion and fission, loss of fragments at the terminal region, alternative splicing, etc., creating complexity in the proteomes of higher eukaryotes including human [4,9-13]. It has been known that the fraction of multi-domain proteins in eukaryotes is about 65% compared to only 40% in prokaryotes [14]. However it is to be noted that evolution in cis-regulatory regions plays a significant role to create the differences in complexity among different species (especially in animals) as they can rapidly produce major changes in gene expression patterns [15].

A general limitation in studying the origins of protein domains is the lack of assigned functional domains for about half of the residues in known proteins in eukaryotic species. Widely used methods for protein domain assignment are: (i) based on three-dimensional structure that include independently foldable units or structural domains and (ii) based on conserved primary sequences that include independently evolving units or conserved functional domains. Despite the differences in the assignment, comparative analysis between these two types of domains revealed overall equivalency in the domain families [16]. A number of resources offer domain information that include structure-based databases, such as SCOP (Structural Classification of Proteins) [17], CATH (Class Architecture Topology Homology) [18], FSSP (Families of Structurally Similar Proteins), [19] etc., or evolutionary-based databases, such as Pfam [20], ProDom [21], SMART [22], etc.. Structure-based domain assignments have been extensively used in the literature [9,23,24] for better understanding of biological functions at the molecular level. Nevertheless, evolutionary-based databases have higher coverage of domains than structure-based databases [14,25].

Domains in protein sequences are basic evolutionary units [17,26] that constantly evolve to attain new functionality either by combining with other domains or by completely changing into a new domain. Sequence-based analyses have demonstrated that some domains have ancient origin with wide spread occurrence in all three kingdoms of life, i.e. archaea, bacteria and eukarya [2], implying their indispensable role in fundamental cellular processes. Thus, many enzymatic domains of central metabolism as well as other non-enzymatic domains appear to owe their heritage to common ancestors in archaea, bacteria and eukarya [8]. On the other hand, newer domain families also emerged in more complex forms of life [27,28] raising one fundamental question: what is the evolutionary origin of these domains? Whether they have emerged from preexisting domain families or novel domain families are generated *ab initio*. Several efforts have been made to solve these questions using comparative analysis of proteomes from different organisms [23,29]. The abundance of protein sequence data available for the entire spectrum of life has prompted us to address these questions for domains in the human proteome. To accomplish this, we have traced back the origin of its constituent domains hierarchically, along the *Homo sapiens *lineage to see at what stage the functional and conserved domains have evolved during the course of evolution. Such understanding of the evolutionary origin of domains helps to elucidate the functional versatility of known proteins as well as the functionality of hypothetical proteins in human. It also helps to understand the divergent evolution of domain families and their degree of divergence to accomplish complex functions in multi-cellular organisms. In this study, we have correlated the functional versatility and evolution of human proteins in the context of its domain evolution. To our knowledge, this is the first report on the origins of functional domains in the entire human proteome carried out by subtractive searching along the evolutionary lineage of *Homo sapiens*.

## Results

**Figure 1:**
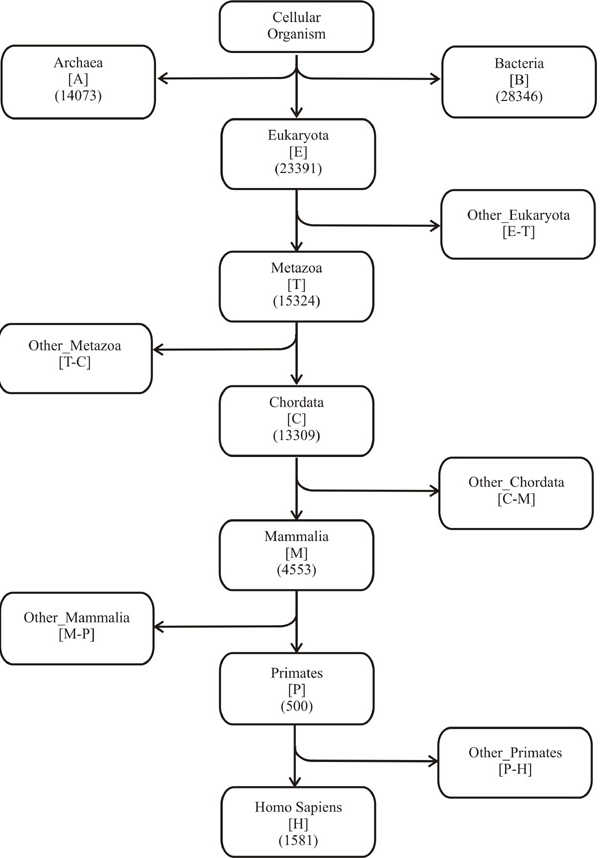
**Taxonomic classification of *Homo sapiens *lineage**. Each central box represents a major node representing a distinct group of species. In each box (other than cellular organism), letter notation for that node is given in square bracket. Each side box (other than archaea and bacteria) was derived by subtracting the sequences from the next higher node from those in the previous lower node. The number of human domains originated at archaea, bacteria and eukaryotic nodes are given in parenthesis. For archaea and bacteria, the number of domains with their remote homologs found both in archaea and bacteria (archaea+bacteria subnode) is 13,052, found only in archaea not in bacteria (archaea_only subnode) is 1,021 and found only in bacteria not in archaea (bacteria_only subnode) is 15,294 (see Methods).

**Figure 2:**
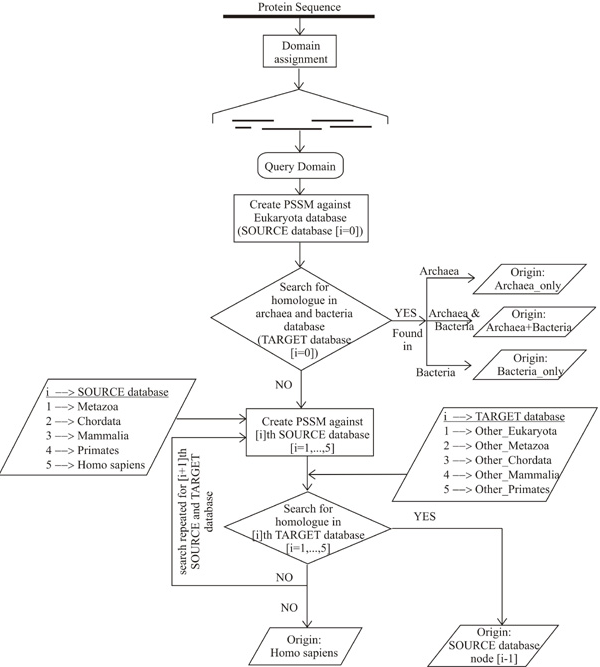
Flow diagram of subtractive searching method depicting the process of tracing the evolutionary origin of human domains.

Figure 1 shows the broad taxonomic classification of the *Homo sapiens *lineage comprising of seven major hierarchical nodes, representing distinct groups of species in the hierarchy. Along these nodes, we have searched for the origin of human protein domains hierarchically, using subtractive searching method illustrated in Figure 2 (see Methods). The results of this study are presented in four sections as follows: First section reports about the origin of human domains at different nodes along the lineage. As each domain is assigned based on Pfam domain definition, corresponding Pfam families can also be distributed over different nodes of origin and the second section describes about the evolutionary distribution of Pfam families along the lineage. Due to the modular nature of proteins, different functional domain architectures in different proteins reflect the functional versatility of those domains as described in section three. Finally, the last section details the patterns of domain origins at different nodes in the context of protein evolution.

### Origin of functional and conserved domains in the human proteome

We have used the HHpred method [30] for identification of functional domains (see Methods) in the human proteome. HHpred employs both sequence and secondary structure-based information in HMM-HMM comparison method and hence, is more sensitive than the 'hmmpfam' search (from HMMER package) [31] in finding remote homologs. As shown in Table 1, HHpred outperformed 'hmmpfam' with 10% more functional domain coverage and 20% higher residue coverage in the human proteome against Pfam-A families. Out of 35,641 protein sequences in the human proteome, 28,190 have been found to contain at least one hit with Pfam families that include 3,853 unique Pfam-A families and 5,149 unique Pfam-B families. Out of 28,190 sequences, 19,252 have matches with Pfam-A families only, 1,971 sequences have matches with Pfam-B only, while, 6,967 sequences have matching domains both with Pfam-A and Pfam-B families.

**Table 1:** Comparison of the performance of HHpred against Hmmpfam in detecting remote homologs

	HHpred method	Hmmpfam method
Pfam-A hits for sequences	74%	64%
Residues covered by Pfam-A hits	54%	34%
Unique Pfam-A families found	3853	3192

Followed by HHpred search, we have obtained 225,360 overlapping domains from 28,190 sequences, each corresponding to at least one Pfam-A or Pfam-B family. Each domain was individually traced for its origin along the *Homo sapiens *lineage, using the subtractive searching method as shown in Figure 2 (see Methods). Because of the high sensitivity of HHpred in detecting functional domains, these domains can be distinct but often, they are partly or fully overlapping (subsets) with each other resulting in different Pfam assignments for overlapping regions. To reduce the overlap, we have merged two or more overlapping domains (within the same node of origin) with either of the boundaries differing by utmost 10 residues (based on the minimum inter-domain linker region) [32], into a new domain. Hence these merged domains have more than one Pfam annotation. Overlapping domains with different nodes of origin (Figure 1) were not merged due to the possibility that a domain may be subjected to different evolutionary pressures in different nodes and hence evolve differently. Ultimately, for 28,190 sequences in the human proteome, we have obtained 88,025 domains, each assigned to at least one Pfam-A or Pfam-B family.

The distribution of human domains (88,025) originated from archaea, bacteria and eukaryotic nodes along the *Homo sapiens *lineage is given in Figure 1. The node of origin for a domain is the first node in the hierarchy where its remote homolog is found. Among domains with prokaryotic origin (~ 33%), bacteria_only subnode (remote homologs first found only in bacteria but not in archaea) covers the highest fraction of 17% (15,294 domains). Among domains with eukaryotic origin, the percentage of new domains originated at different nodes of origin gradually diminishes from nodes eukaryota (27%, 23,391 domains) to primates (1%, 500 domains). Finally, only 1,581 domains (~ 2%) were found to have originated at the *Homo sapiens *node. These domain distributions suggest that about 60% of human domains have their origins at very early stages of evolution (archaea, bacteria and eukaryota nodes) before the metazoan era. A gradual reduction in the origin of new domains in higher nodes also suggests that proteins in the higher forms of life have evolved mostly by reusing existing domains from the protein repertoire rather than acquiring completely new domains [9].

### Evolutionary distribution of Pfam families assigned to the human proteome

**Figure 3:**
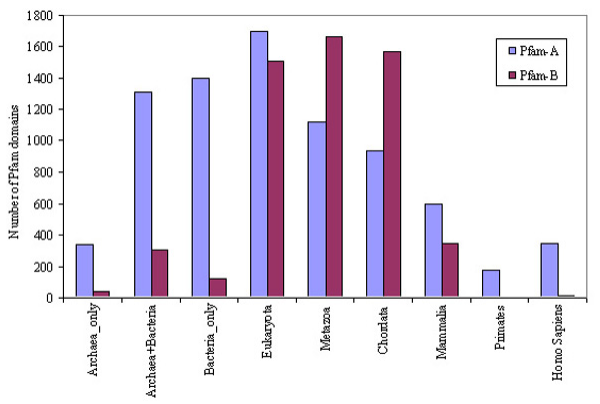
Number of unique Pfam-A and Pfam-B families associated with human domains (redundant across nodes) with origin at different nodes.

All the human domains identified in this study have at least one annotation with either Pfam-A or Pfam-B families. Based on the nodes of origin of human domains, we have mapped the node-wise distribution of corresponding Pfam families (Figure 3). Most of the Pfam-A families are found in bacteria, archaea and eukaryota nodes, implying that most of the known functions in the human proteome were emerged at very early stages of evolution. Alternatively, this could be partly due to the fact that domains appearing in the early stages of evolution have been well characterized in the literature compared to those seen at the later stages. On the contrary, a vast majority of Pfam-B families (conserved families with no functional annotation) are found at eukaryota, metazoa and chordata nodes indicating that these newer functionalities have evolved with the lower eukaryotic species. Interestingly, very few unannotated families (Pfam-B) have their origin at primates and *Homo sapiens *nodes. Nevertheless, it should be noted that the assigned Pfam-A or Pfam-B families cover only 79% of proteins and 67% of residues in the human proteome and the remainder could possibly contain new domains specific to higher eukaryotic species.

#### Trends in domain evolution

**Figure 4:**
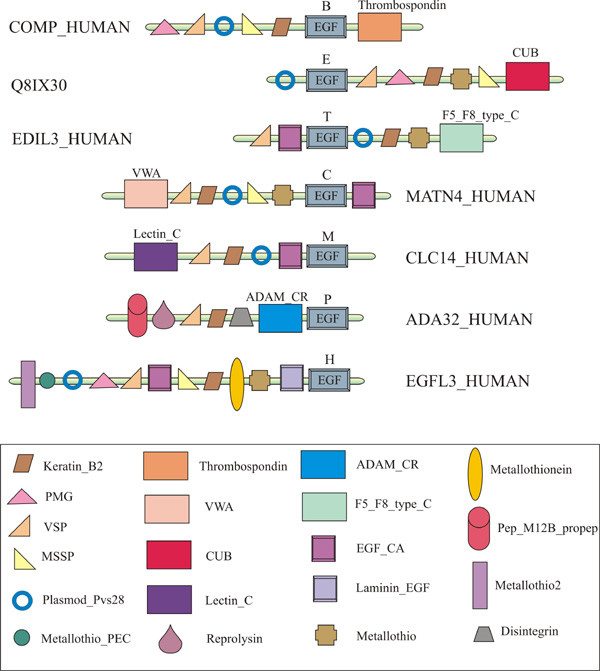
**Cartoon diagram of different representative proteins containing Pfam-A family EGF (epidermal growth factor) with remote homologs found at different nodes along the lineage using subtractive searching method**. For each sequence, SWISS-PROT identifier is given and EGF domain is shown along with the node name where it has found its remote homolog in that protein sequence. The codes for different nodes are: B, bacteria; E, eukaryota; T, metazoa; C, chordata; M, mammalia; P, primates; H, *Homo sapiens*. Other functionally significant domain names in protein sequences are given in the legend.

**Figure 5:**
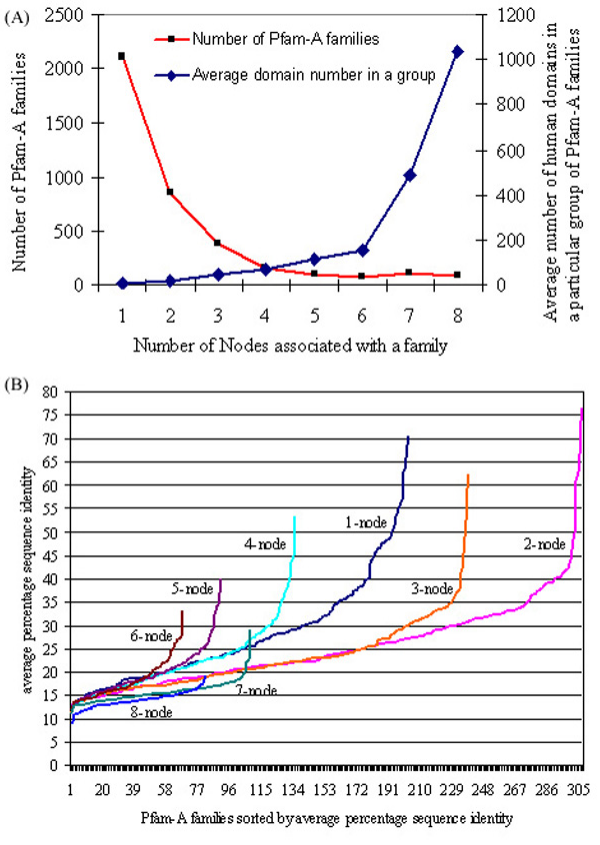
**Grouping of Pfam-A families according to the number of nodes (where remote homologs are found along the lineage) associated with it**. (A) Distribution of different groups of Pfam-A families is plotted in the left axis with decaying nature best approximated by power-law with Y ~ X^-1.7 ^(R^2 ^= 0.95), compared to an exponential, linear or logarithmic function, whereas average numbers of human domains in each group of Pfam-A families are plotted in the right axis with exponentially rising nature with Y = 3.6 × e^-0.7X ^(R^2 ^= 0.97). (B) Distribution of sorted Pfam-A families by average percentage sequence identity of domains within same family (families with number of domains less than 10 are excluded from this graph) in different groups. The maximum probable range of each curve is the more flattened portion.

Ideally, each unique Pfam-A family should find remote homologs only at one node (the node of origin) since we have used the subtractive searching method (Figure 2) for detecting the origin of domains. Nevertheless, we have used a very sensitive HMM-HMM comparison method for annotating human domains but searched for the node of origin of those domains using a sequence-profile based method. Highly diverse Pfam-A families (often members of a clan) have found their remote homologs at multiple nodes since the sequence diversity of such families is beyond recognition by the profile-based method (PSI-BLAST) used in this study. Figure 4 illustrates such an example of Pfam-A family, EGF (epidermal growth factor), an important building block in numerous extracellular matrix proteins including growth factors, transmembrane receptors, and soluble secreted proteins. While the node of origin for EGF family is bacteria, its remote homologs have been found at different nodes along the lineage in different proteins using the subtractive searching method. Since domain evolution and protein evolution are interdependent, well-distributed families are expected to show higher diversity resulting in their identification in multiple nodes. We have tested this hypothesis as follows. First, we grouped all Pfam-A families assigned to human domains based on the number of nodes where their remote homologs were found such as: 1-node, 2-node ... up to 8-node. Then we counted the frequency of human domains belonging to each Pfam-A family and calculated the average number of human domains in each group of families. Figure 5A demonstrates contrasting behavior between the number of Pfam-A families in each group and the average number of human domains in those groups, suggesting that the number of Pfam-A families gets smaller (decaying curve) and the size of Pfam-A families gets larger (rising curve), with increasing number of nodes where their remote homologs are found. The decaying curve depicts the relationship between the number of functional families and degree of divergent evolution for a family which is best approximated by a power-law (which means few functional families undergo high degree of divergent evolution), where Y ~ X^-1.7^, with R^2 ^= 0.95. The rising curve represents the number of human domains in such divergent functional families which is best approximated by exponential decay curve with Y ~ e^-0.7X ^(R^2 ^= 0.97).

We have further calculated the average global sequence identity among human domains belonging to the same Pfam-A family, as sequence identity is generally regarded as an inverse metric of sequence diversity. Figure 5B shows the group-wise distribution of Pfam-A families sorted by average percentage sequence identities among their domains. The lower limits for all groups generally start below 15%, except in 7-node and 8-node groups with average sequence identities lower than 10%. Similarly, the upper limits gradually decrease as the number of nodes goes up indicating that families with remote homologs at more nodes show decreasing range of sequence identities among their members. In other words, if a functional family finds remote homologs in 8 nodes, it has gone through the highest degree of divergent evolution as measured by the sequence identity among its members. Hence, the number of nodes associated with different groups of Pfam-A families indicate the degree of divergence of that family during the course of evolution. Correlating both Figures 5A and 5B, we can conclude that abundant functional families undergo higher degree of divergent evolution but, they are less frequent. Table 2 reports some of the functionally known Pfam-A families in each group in Figure 5A. One can observe the presence of functional families related with more diverse functions (such as immunoglobulin domain, zinc-finger domain, protein kinase, ankyrin repeat, SH3 domain, 7 transmembrane receptors, epidermal growth factors, etc.) at multiple node groups.

**Table 2:** Some of the functionally known Pfam-A families in each group, defined by the number of nodes associated with it

Number of nodes associated with a group	Pfam-A family ID	Frequency of occurrence in human proteome	Description of the family
1	PF02214	120	K+ channel tetramerization domain
	PF02101	113	Ocular albinism type 1 protein
	PF02719	112	Polysaccharide biosynthesis protein
	PF00307	109	Calponin Homology domain
	PF04185	19	Phosphoesterase family
			
2	PF02117	671	C. *elegans *Sra family integral membrane protein
	PF04762	306	IKI3 family
	PF00089	175	Trypsin
	PF00854	159	Proton-dependent oligopeptide transport family
	PF00969	133	Class II histocompatibility antigen, beta domain
			
3	PF01748	923	C. *serpentine *receptor like protein
	PF05462	883	Slime mold cyclic AMP receptor
	PF00002	862	7 transmembrane receptor (secretin family)
	PF03125	791	C. *elegans *Sre G protein-coupled chemoreceptor
	PF02118	777	C. *elegans *Srg family integral membrane protein
	PF00169	307	Pleckstrin homology domain
	PF02175	305	C. *elegans *integral membrane protein Srb
	PF07653	273	Variant SH3 domain
			
4	PF01461	900	7 transmembrane chemoreceptor
	PF03402	642	Vomeronasal organ pheromone receptor family
	PF01163	461	RIO1 family
	PF01352	461	Kruppel-associated box domain
	PF00076	313	RNA recognition motif
	PF00018	288	SH3 domain
	PF00046	276	Homeobox domain
	PF00595	210	PDZ domain
			
5	PF00096	1007	Zinc finger, C2H2 type
	PF05296	917	Mammalian taste receptor protein (TAS2R)
	PF00047	834	Immunoglobulin domain
	PF00069	769	Protein kinase domain
	PF08205	647	CD80-like C2-set immunoglobulin domain
	PF07679	621	Immunoglobulin I-set domain
	PF00071	317	Ras family
			
6	PF03326	1403	Herpes virus transcription activation factor
	PF07686	965	Immunoglobulin V-set domain
	PF07714	767	Protein tyrosine kinase
	PF04388	713	Hamartin protein
	PF07654	489	Immunoglobulin C1-set domain
	PF00131	344	Metallothionein
	PF00023	310	Ankyrin repeat
	PF00041	261	Fibronectin type III domain
			
7	PF05109	2177	Herpes virus major outer envelope glycoprotein
	PF03546	1859	Treacher Collins syndrome protein Treacle
	PF00038	1194	Intermediate filament protein
	PF00001	1004	7 transmembrane receptor (rhodopsin family)
	PF01391	277	Collagen triple helix repeat
	PF00008	221	Epidermal Growth Factor-like domain
			
8	PF03154	3365	Atrophin-1 family
	PF04554	3125	Extensin-like region
	PF03251	2546	Tymo virus 45/70kd protein
	PF05956	2384	APC basic domain
	PF01500	766	Keratin, high sulfur B2 protein

#### Origin of functional families

**Figure 6:**
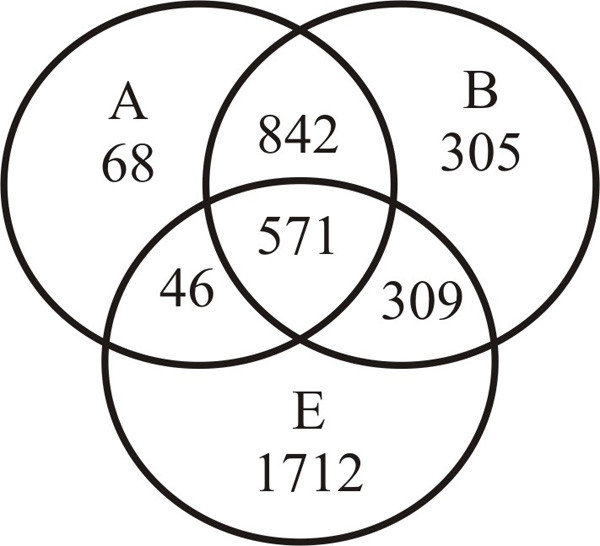
**Distribution of Pfam-A families according to the origin in three kingdoms of life – archaea, bacteria and eukaryota**. The codes for different nodes are: A, archaea; B, bacteria; E, eukaryota; E here represents eukaryota node and all nodes above it.

Since we have used the subtractive searching method, ideally, all human domains assigned to a Pfam-A family should detect their remote homologs only at a single node, which is considered the origin of that family. Nevertheless, about 45% of Pfam-A families (1,741) have their remote homologs from multiple nodes for reasons explained in the above paragraph. In these cases, the origin of a functional family is at the lowest node where a remote homolog was first found along the *Homo sapiens *lineage. Nevertheless, no hierarchy was applied between archaea and bacteria. The distribution of Pfam-A families assigned to human domains among three kingdoms of life is illustrated using a Venn diagram in Figure 6, while Table 3 provides the data for evolutionary origin of Pfam-A families in different eukaryotic nodes. Out of 3,853 unique Pfam-A families observed in human domains, ~ 56% (2,141 families) have prokaryotic origin (Figure 6) of which, 926 families are also shared in eukaryotic species. Among families with eukaryotic origin, the highest number of unique families is exclusive to the eukaryota node (1001, ~ 26%), with 667 (~ 17%) families originated only at eukaryota node which could explain the number of newer functions acquired by early eukaryotes to make the transition from their simpler prokaryotic ancestors. The next highest number of families are associated with metazoans (370, ~ 10%) and ultimately there are 44 (~ 1.14%) Pfam-A families associated with only *Homo sapiens *node. Pfam-A families with multiple nodes of origin are mostly associated with archaea and bacteria while their numbers decreased gradually down the hierarchy. From Figures 4, 6 and Table 3 we can see that a vast majority of the protein domain repertoire has evolved between the nodes eukaryota to chordata and the combination of these domains rather than the new domains are significant (supporting previous analyses) [9] for the functional diversity of proteomes from the mammalian node and onwards.

**Table 3:** Evolutionary origin of Pfam-A families at different eukaryotic nodes

Node of origin	Number of Pfam-A families with remote homologs at		
	
	Single node	Multiple node	Total
Eukaryota	667	334	1001
Metazoa	255	115	370
Chordata	154	64	218
Mammalia	54	18	72
Primates	4	3	7
*Homo sapiens*	44	0	44

Table 4 reports some of the most frequent Pfam-A families with origin at different evolutionary nodes. In the archaea node, functions related with fundamental processes of life such as ribosomal proteins, RNA and DNA polymerases, translation initiation factors, etc. are prominent. In addition to these, bacterial node showed more enzymatic functions where sulfotransferase family appeared with high frequency. In the eukaryotic node, ion-channel related families, calponin homology domain, eukaryotic vesicle transport processes (involved in secretory pathway), motor proteins of eukaryotic cells, etc. – all characteristic of eukaryotes start to emerge. Metazoans are multicellular animals having cells differentiated into tissues and organs with distinct nervous system. Several functions related to the development of nervous system such as wnt, T-box, FEZ, ephrin, sema were seen for the first time at the metazoan node. The chordata node includes the characteristics of invertebrates as well as vertebrates, though vertebrate-specific functions are more observable at this node. Connexins, gap junction protein forming hemichannels, found only in vertebrates, are first found at chordata node. Other frequently found functions are related with immune system (interferon, MHC_II_alpha, etc.) and cell adhesion (protocadherin, fibronectin, etc.). Appearance of less frequent, but characteristic vertebrate-specific functions (based on Pfam annotation) at chordata node is noteworthy, like, functions associated with the development of vertebrate nervous system, regulating microtubules during mitotic metaphase, mature olfactory sensory receptor neurons, etc. Mammalians are characterized by the presence of mammary glands and several mammalia-specific functions are evolved at this node. These functions include casein – major milk protein, mammalian apolipoprotein CIII sequences, immunoglobulin C2-set domain – present in mammalian T-cell surface antigen CD2 proteins, etc. There are very few Pfam-A families which are first seen at the primates node. Of these, the noteworthy is SPAN-X which is a cancer-testis antigen and potential target for cancer immunotherapy. Lastly at the *Homo sapiens *node, Pfam-A families related to viral diseases or proteins with unknown function such as, L1 Late protein, Early protein E2_N, E6, E7; GP120; Fusion_Gly; peptidase_C3, BAGE – B melanoma antigen family, etc. are more prevalent. The BAGE gene encodes a human tumor antigen that is recognized by a cytolytic T lymphocyte. The high abundance of viral disease-related Pfam-A families associated with *Homo sapiens *node may be explained by the horizontal gene transfer from viruses to human. Thus, it is interesting to note how species-specific functions are originated at different stages of life, reflecting increased functional complexity from unicellular lower organisms to multicellular higher organisms.

**Table 4:** Some frequently populated Pfam-A families with origin at different evolutionary nodes.

Pfam-A family	N*	Functional description
Archaea_only (131 sequences)		

Ribosomal proteins	25	Involved in catalyzing mRNA-directed protein synthesis
RNA polymerase	20	Catalyse the DNA dependent polymerisation of RNA
Translation initiation factor	12	Required for maximal rate of protein biosynthesis, in directing ribosome to proper start state of translation
DNA polymerase	5	Required in replication of DNA
Diphthamide_syn	5	Putative diphthamide synthesis protein

Bacteria_only (1102 sequences)		

Sulfotransferases	67	Responsible for the transfer of sulphate groups to specific compounds
Tubulin	35	Major component of microtubules, involved in polymer formation
DAGAT	23	The enzyme diacylglycerol acyltransferase involved in the catalysis of terminal step of triacylglycerol
Carb_anhydrase	23	Carbonic anhydrase, catalyze reversible hydration of carbon dioxide
2OG-FeII_Oxy	21	2-oxoglutarate and Fe(II)-dependent oxygenase superfamily
Ribosomal protein	17	Involved in catalyzing mRNA-directed protein synthesis

Eukaryota (2928 sequences)		

K_tetra	120	K+ channel cytoplasmic tetramerisation domain
Ocular_alb	113	X-linked disorder characterized by severe impairment of visual acuity, retinal hypopigmentation and the presence of macromelanosomes
CH	109	Calponin homology domain, found in both cytoskeletal and signal transduction protein
Histone	68	Core Histone H2A/H2B/H3/H4, involved in histone-histone and histone-DNA interactions
7 tm_3	65	7 transmembrane receptor (metabotropic glutamate family), coupled to G-proteins and stimulate the inositol phosphate/Ca^2+ ^intracellular signalling pathway
Actin	62	Involved in formation of filament, major component of cytoskeleton
Fork_head	59	A transcription factor that promotes terminal rather than segmental development, involved in early developmental decisions of cell fates during embryogenesis
UQ_con	59	Ubiquitin-conjugating enzyme, involved in catalytic activity or assist in poly-ubiquitin chain formation

Metazoa (1362 sequences)		

Zf-C4	88	DNA binding domain of a nuclear hormone receptor
PID	67	Phosphotyrosine interaction domain
RA	51	Ras association domain
sema	48	The Sema domain occurs in semaphorins, which are a large family of secreted and transmembrane proteins, some of which function as repellent signals during axon guidance
Ets	37	Erythroblast transformation specific domain, required for induction of erythroblastosis
Wnt	25	Role in intercellular communication, possible role in central nervous system
T-box	24	Perform DNA-binding and transcriptional activation/repression roles

Chordata (470 sequences)		

Connexin	22	Gap junction protein
Interferon	19	Produce antiviral and antiproliferative responses in cells
Protocadherin	17	Cadherin-related molecules in central nervous system
MHC_II_alpha	15	Related with cell-mediated immune responses
Fn2	14	Fibronectin type II domain, involved in a number of important functions e.g., wound healing; cell adhesion; blood coagulation; cell differentiation and migration; maintenance of the cellular cytoskeleton; and tumour metastasis

Mammalia (146 sequences)		

Gag_p10	21	The p10 or matrix protein (MA) is associated with the virus envelope glycoproteins in most mammalian retroviruses and may be involved in virus particle assembly, transport and budding
GP41	16	The GP41 subunit of the envelope protein complex mediates membrane fusion during viral entry
Bim_N	11	Bim protein N terminus, essential initiators of apoptotic cell death

Primates (21 sequences)		

SPAN-X	14	Human sperm proteins associated with the nucleus and mapped to the X chromosome, they are cancer-testis antigens.

*Homo sapiens *(120 sequences)		

GP120	9	Envelope glycoprotein GP120
BAGE	5	B melanoma antigen family

* N is the number of protein sequences containing those Pfam-A families in the left column.

### Functional versatility of domains in the human proteome

**Figure 7:**
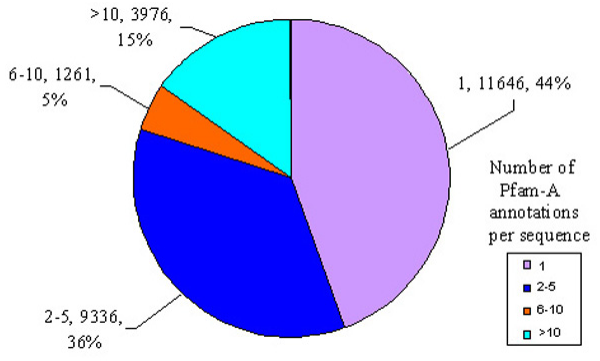
**Distribution of protein sequences according to the total number of Pfam-A annotations in each**. For each category, the number of Pfam-A annotations in a sequence, followed by the total number of sequences under that category are given along with the percentage of sequences in each category with respect to the total number of sequences with Pfam-A hits.

To identify the number of unique functions conferred by the constituent domains of a protein, related Pfam-A families were grouped together according to Pfam clans as they were thought to have the same evolutionary origin [33]. In each full-length sequence, we counted the number of unique Pfam-A families/clans which reflects the general functional complexity of a protein (Figure 7). In the human proteome, about 26,219 sequences (~ 74%) have matches with at least one Pfam-A family. Out of these, about 44% have only one Pfam-A annotation, ~ 36% have 2–5 annotations, ~ 5% have 6–10 annotations while a surprising 15% have more than 10 annotations (Figure 7). Proteins belonging to the last category include functionally diverse enzymes, structural families, and a large number of virus-related pfam-A families.

To better understand the functional versatility of human domains, we have analyzed the frequency of individual functional families in proteins with increasing order of functionalities i.e., proteins containing 1 to 5 different Pfam-A annotations. Table 5 lists the top 10, most frequent Pfam-A families/clans in human protein sequences containing 1–5 Pfam-A annotations. Among protein sequences with only one Pfam-A annotation, the most commonly found family/clan is immunoglobulin superfamily (Ig) including member families such as V-set, I-set, C1-set, C2-set, etc. The other two most frequent families in this category are zinc finger family and protein kinase superfamily. Zinc finger family becomes the most frequently found in protein sequences with two functional annotations, followed by kruppel-associated box and Ig superfamily. Among those with three Pfam-A annotations, G-protein superfamily becomes the most prominent while Ig superfamily again leads the group with four Pfam-A annotations. Moreover, the Ig clan is one of the top 4, most frequent families from all groups except the group with 5 annotations. Also, Atrophoin-1 family which is associated with DRPLA disease (Dentatorubral pallidoluysian atrophy or Smith's disease) is a frequent member of proteins with four or five functional annotations. In the group with five functional annotations, a strikingly high frequency observed in the first five families/clans (Table 5) is noteworthy. These families include Frizzled superfamily, family-A G-protein coupled receptor-like superfamily, mammalian taste receptor proteins, *C. elegans *chemosensory receptor and C. *elegans *Srg family integral membrane proteins. Interestingly, the origin of all these domains is either at eukaryota or at metazoa nodes. Frizzled proteins function in multiple signal transduction pathways and are essential for embryonic development [34]. It is also noteworthy that domains corresponding to three families/clans – Ig superfamily, zinc finger proteins and protein kinase superfamily were among the most frequent families in protein sequences with one, two and three functional annotations. This suggests that these domains can carry out essential functions as standalone domains and also extend their functionality to accomplish complex tasks in combination with one or more other domains.

**Table 5:** Most frequent Pfam-A families/superfamilies in protein sequences that are associated with single or multiple functions

F^a^	P^b^	T^c^	Most abundant families/superfamilies (Top 10)	
			
			Description	N^d^
1	11,646	1,778	Immunoglobulin superfamily (Ig)	598
			Zinc finger family (Zf-C2H2)	348
			Protein kinase superfamily (Pkinase)	339
			FAD/NAD(P)-binding Rossmann fold Superfamily (NADP_Rossmann)	191
			Ankyrin repeat	151
			RNA recognition motif (RRM_1)	130
			Major Facilitator Superfamily (MFS)	114
			Ig-like fold superfamily (E-set)	112
			Peptidase clan PA	106
			Methyltransferase superfamily	104

2	4,933	1,333	Zinc finger family (Zf-C2H2)	477
			Kruppel-associated box	326
			Immunoglobulin superfamily (Ig)	322
			Marek's disease glycoprotein A	206
			WD-40 repeats (beta-transducin repeats)	167
			Protein kinase superfamily (Pkinase)	160
			Ig-like fold superfamily (E-set)	150
			IKI3 family	149
			FAD/NAD(P)-binding Rossmann fold Superfamily	124
			POZ domain superfamily	99

3	2,229	965	G-protein superfamily	166
			G-protein alpha subunit	136
			Dynein light intermediate chain	132
			Immunoglobulin superfamily (Ig)	113
			WD-40 repeats (beta-transducin repeats)	104
			Zinc finger family (Zf-C2H2)	98
			IKI3 family	96
			Keratin, high sulfur B2 protein	96
			Protein kinase superfamily (Pkinase)	93
			Zinc finger, C3HC4 type (Ring finger)	90

4	1,038	678	Immunoglobulin superfamily (Ig)	90
			Atrophin-1	84
			Keratin, high sulfur B2 protein	83
			Class II histocompatibility antigen, beta domain	77
			Class I histocompatibility antigen, domain alpha 1 and 2	77
			Extensin-like region	71
			Giardia variant-specific surface protein	67
			Class I histocompatibility antigen, C- terminus	66
			P-loop containing nucleoside triphosphate hydrolase superfamily	57
			Family A G protein-coupled receptor-like superfamily	56

5	1,136	536	Frizzled/OA1/CAR/Secretin receptor-like superfamily	676
			Family A G protein-coupled receptor-like superfamily	675
			Mammalian taste receptor protein	664
			*C. elegans *chemosensory receptor	660
			*C.elegans *Srg family integral membrane protein	659
			Atrophin-1	97
			Keratin, high sulfur B2 protein	92
			Giardia variant-specific surface protein	77
			Extensin-like region	63
			Dentin matrix protein 1	53

^a ^F = Number of functions associated with a protein sequence

^b ^P = Number of protein sequences associated with F number of functions

^c ^T = Number of unique Pfam-A families associated with P number of protein sequences

^d ^N = Frequency of a Pfam-A family in P number of protein sequences

We have further investigated the occurrence frequency of functional domain combinations in human proteins, as it is interesting to note which combinations are prevalent over the others. A number of Pfam-A domains detected in our study are not reported in the UniProt annotation for individual proteins. HHpred method used in this study has enabled us to assign more functional domains to human proteome (Table 1) and consequently makes the analysis described in this section more meaningful. Table 6 reports some commonly occurring domain combinations (not ordered) in the protein sequences with more than one Pfam-A annotation. The most abundant combination in the proteins with two Pfam-A annotations is the zinc-finger (zf-C2H2) family with Kruppel-associated box (KRAB), which are mainly involved in transcriptional regulation [35,36]. The next most frequent combination is Ig superfamily with Marek's disease glycoprotein A. These proteins are mainly glycoprotein precursors or immunoglobulin-like receptors containing immunoglobulin-like domains. UniProt annotations for these proteins do not show Marek's disease glycoprotein-A annotation. However, immunoglobulin gene superfamily is thought to play an immunoevasive role in the pathogenesis of Marek's disease mainly found in birds [37]. Similarly, protein sequences containing IKI3 and WD-40 families correspond to various functions such as substrate selectivity, catalytic activity, development in peripheral and central nervous system, etc. [38,39]. The most abundant combination in the proteins with three-domain architecture is G-protein alpha subunit, G-protein superfamily and dynein light intermediate chain. These are mainly GTP-binding proteins and Ras-related inhibitors of cell growth. Another frequently found combination in this category includes major histocompatibility antigen (MHC) class-I, class-II beta domain and immunoglobulin superfamily, which are mainly found in class II histocompatibility antigen, beta chain precursor proteins. Similarly, in several Kelch-like proteins, frequently found domain combinations are POZ domain superfamily, recombination activating protein and Kelch repeats. These proteins are involved in many aspects of cell function, such as actin-associated proteins, cell morphology and organization, gene expression, viral binding partners and have extracellular roles [40]. Histocompatibility antigen proteins, found in three-domain combinations, are again found in sequences with four domain combinations with an additional family, C-terminal region of class I histocompatibility antigen. These proteins are mainly class-I histocompatibility antigen, alpha chain precursor proteins. Another frequently found domain combination in this category include ATPase proteins with cation transporter/ATPase N-terminus, E1–E2 ATPase, haloacid dehalogenase superfamily and cation transporter C-terminus, which are known to play a crucial role in ion transportation across biological membranes [41]. A large fraction (~ 58%) of the protein sequences among the sequences with five domain architecture (1136 sequences, shown in Table 5) include the domain combination of (i) C. *elegans *chemosensory receptor superfamily, (ii) C. *elegans *Srg family integral membrane protein, (iii) Frizzled/OA1/CAR/Secretin receptor-like superfamily, (iv) mammalian taste receptor protein family and (v) Family A G protein-coupled receptor-like superfamily. Similar combination without Srg family is found in proteins with four domain combinations, but in much less frequency compared to this five domain combinations. These proteins are part of the well-known G-protein-coupled receptors superfamily with highly diverse structure and function, and are highly abundant in C. *elegans *[42].

**Table 6:** Some commonly occurring functional domain combinations (not ordered) in protein sequences with multiple Pfam-A annotations

F^a^	Different domain combinations	N^b^
2	Kruppel-associated box & Zf-C2H2 zinc finger family	316
	Immunoglobulin superfamily & Marek's disease glycoprotein A	206
	IKI3 family & WD-40 repeats	149

3	G-protein alpha subunit & G-protein superfamily & Dynein light intermediate chain	131
	MHC_I & MHC_II_beta & immunoglobulin superfamily	50
	POZ domain superfamily & recombination activating protein 2 & kelch repeat superfamily	50

4	MHC_I & MHC_II_beta & immunoglobulin superfamily & MHC_I_C	66
	Cation transporter/ATPase, N-terminus & E1-E2 ATPase & haloacid dehalogenase (HAD) superfamily & Cation transporter/ATPase, C-terminus	38
	*C. elegans* chemosensory receptor & Frizzled/OA1/CAR/Secretin receptor-like (FOCS) superfamily & mammalian taste receptor protein(TAS2R) & Family A G protein-coupled receptor-like superfamily	36

5	*C. elegans* chemosensory receptor &*C.elegans* Srg family integral membrane protein & Frizzled/OA1/CAR/Secretin receptor-like (FOCS) superfamily & mammalian taste receptor protein(TAS2R) & Family A G protein-coupled receptor-like superfamily	659

^a ^F = Number of functions associated with a protein sequence

^b ^N = Number of occurrences of corresponding combination

### Proteome-wide patterns of nodes of origin in human proteins

**Figure 8:**
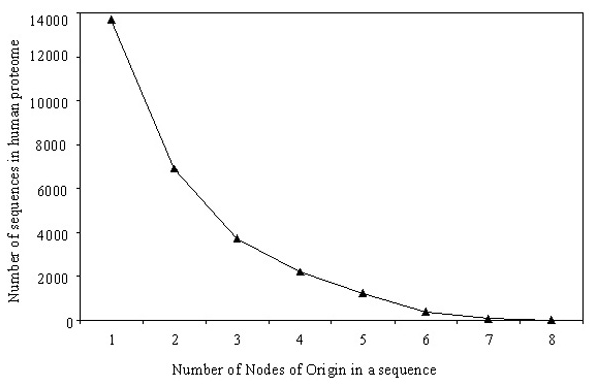
**Graph of the distribution of protein sequences in single or multi node combinations**. This curve can be best approximated by exponential decay curve [Y = 2.6 × 10^4^.e^-0.65X ^with R^2 ^= 0.99].

Analyses of origin of domains at the protein level reveal that domain(s) within a human protein sequence may originate from a single node or from multiple nodes along the evolutionary lineage. Protein sequences are grouped according to the number of nodes of origin of its constituent domains to show the distribution of these groups in the human proteome (Figure 8). We have found a spectrum of proteins with multiple nodes of origin, while only one sequence claimed a maximum of eight nodes of origin. Figure 8 follows an exponential decay curve [Y = 2.6 × 10^4^.e^-0.65.X ^with R^2 ^= 0.99], implying that very few proteins can sustain such intense evolutionary pressure of acquiring new domains at each node.

**Figure 9:**
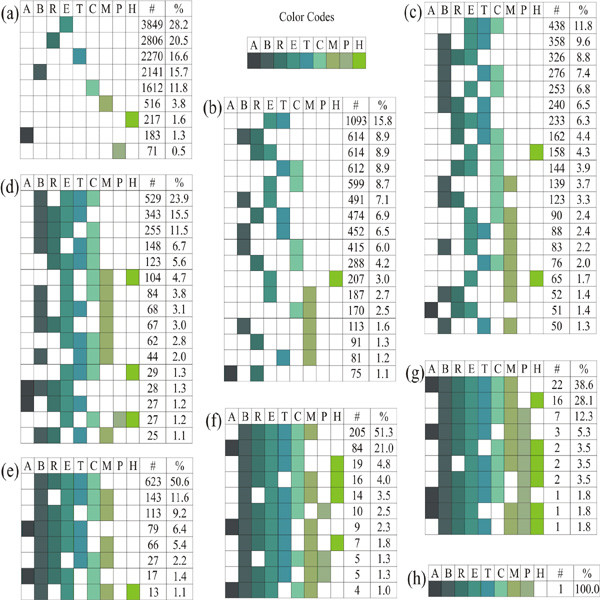
**Different patterns of nodes of origin for protein sequences grouped according to the number of nodes of origin of its constituent domains**. The codes for different nodes are: A, archaea; B, bacteria; R, archaea+bacteria; E, eukaryota; T, metazoa; C, chordata; M, mammalia; P, Primates; H, *Homo sapiens*. Different groups of protein sequences are: (a) 1-node combination, (b) 2-node combination, (c) 3-node combination, (d) 4-node combination, (e) 5-node combination, (f) 6-node combination, (g) 7-node combination and (h) 8-node combination. In each group, number of colored boxes in each row represents the number of node combinations present in each protein sequence under that group, where the number of protein sequences in that node combination is given in the column denoted by '#' and percentage of those sequences out of total sequences in that group is given in the column denoted by '%'. Total number of sequences in each group with different node combinations is given in Figure 8.

Figure 9 illustrates the patterns of nodes of origins for proteins in each group in Figure 8. The constituent domains of a large fraction of human proteins (13,665, ~ 38.5%) have their origins at a single evolutionary node (number of protein sequences for each node are shown in Figure 9a) out of which, 217 sequences have originated only from the *Homo sapiens *node. Out of these single node sequences, most protein sequences (36.2%) have their origin from bacteria (bacteria_only (B) plus archaea+bacteria (R)) followed by the eukaryotic node (28.2%). Careful study of the functions of proteins containing domains first seen at a specific node reveals how basic functions have evolved into complex ones from unicellular to multicellular organisms (as shown in Table 4). Proteins with domains originating from last three nodes- mammalia, primates and *Homo sapiens *are fewer indicating that more domain recombinations have evolved rather than newer domains in higher organisms. These protein sequences are generally single domain proteins (one Pfam-A family covering the full length of the protein) or some have single known domains (a segment of the protein assigned to Pfam-A or Pfam-B). Examples of new domains emerged at these higher nodes include ApoA-II, IL2, Resistin, SPAN-X, BAGE, etc.

**Figure 10:**
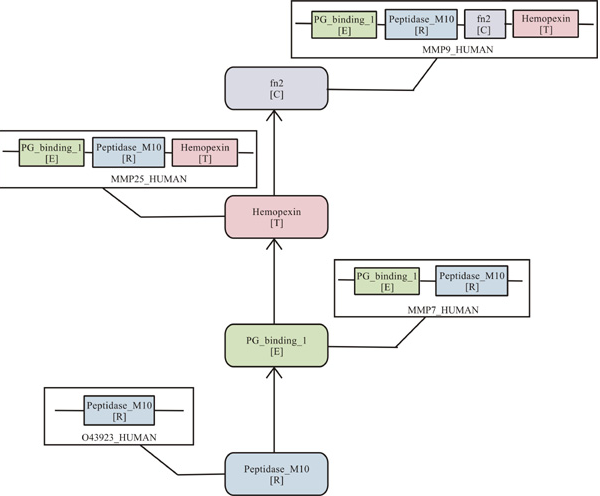
**Cartoon diagram showing the evolution of metalloproteinase family through different stages of evolution**. Each central box represents the insertion of a new Pfam-A domain at different evolutionary nodes shown in square brackets. The codes for different nodes are: R, archaea+bacteria; E, eukaryota; T, metazoa; C, chordata. SWISS-PROT identifiers for human protein sequences are given in side boxes along with their domain compositions.

Since a vast majority of human domains (~ 77%) originate from species early in the evolutionary lineage such as bacteria, archaea, eukaryota and metazoa (Figure 3), these nodes are the most visible in proteins with multi-node origin, such as eukaryota-metazoa combination (Figure 9b), eukaryota-metazoa-chordata combination (Figure 9c), bacteria-eukaryota-metazoa-chordata combination (Figure 9d), and so on. In these combinations, generally the domains with origin at higher nodes are assigned to Pfam-A families which have already found their homologs at the lower nodes (Pfam-A families with homologs at multiple nodes). We have noticed the high occurrence of Pfam-B families among eukaryota, metazoa and chordata nodes (Figure 3), which are part of many proteins in the multi-node combinations. Another important observation in these patterns of nodes in proteins with multi-node combination (Figure 9) is that lower nodes, like, bacteria, eukaryota, or metazoa, are almost always present in 5-, 6- or 7- node combinations. All these observations support the fact that complex proteins have evolved by extending the functionality of existing domains by insertion, deletion or recombination of domains in the protein repertoire [4,9]. In contrast, when entirely new functionalities emerge in higher nodes, they generally appear from completely new proteins, not in combination with existing domains. Here, we present one such example on the evolution of human metalloproteinase proteins (Figure 10) where new domains are added at different stages of evolution to cope up with the increased complexity in protein functions.

Metalloproteinase proteins generally have metalloendopeptidase activity which catalyses the hydrolysis of non-terminal peptide linkages in oligopeptides or polypeptides. Enzymes of this class contain a chelated metal ion essential to their catalytic activity at their active sites. O43923_HUMAN is such metalloproteinase protein with single known domain, peptidase_M10, with its origin at archaea+bacteria node. This peptidase_M10 domain is found in combination with peptidoglycan binding domain (PG_binding_1) of eukaryotic origin at its N-terminal end in MMP7_HUMAN (matrilysin precursor/matrix metalloproteinase-7). Peptidoglycan binding domain is generally involved in the bacterial cell wall degradation with general peptidoglycan binding function [43]. Keeping this combination of domains intact, hemopexin domain, of metazoan origin, at the C-terminal end of the protein is found in MMP25_HUMAN, matrix metalloproteinase-25 precursor. Hemopexin is a serum glycoprotein that binds to haem and transports it to the liver for breakdown and iron recovery, after which the free hemopexin returns to circulation, it prevents haem-mediated oxidative stress. In MMP25_HUMAN, hemopexin domain has been shown to facilitate binding and denaturation of the macromolecular substrates [44]. In MMP9_HUMAN, a matrix metalloproteinase-9 precursor/gelatinase B [45], fibronectin type II domain was inserted at the chordate node, along with all other previously mentioned domains. This domain is found to be responsible for the elastase activity of the protein [46] and involved in binding to gelatin [44], unique to gelatinases. The evolution of human metalloproteinases shows that addition of new domains at different stages of evolution extends the functionality of proteins while preserving their core functionality.

## Discussion

Whether protein structure and/or function space is continuous, or evolutions leap to create new functions in this universe – these are long sought questions over the decade [1,47]. These questions are addressed repeatedly by several researchers in several ways – either by creating phylogenetic trees of life, or by analyzing domain organizations/combinations in different proteomes from different organisms [8,10,23,29,48]. Here, we address these questions by tracing the evolutionary origins of constituent domain in the human proteome.

In this report, assignment of functional domains was carried out by a very sensitive method HHpred [30], which uses both sequence and secondary structural information in HMM-HMM comparison method. The useful outcomes of this method include: (i) better functional space coverage in human proteome compared to regular hmmpfam method and (ii) better detection of remote homologs beyond the capacity of simple profile-sequence comparison method, especially for those families where structure diverges more slowly than sequence [49]. In this study, we came across several instances where functional annotation of a domain by HHpred is not observed in UniProt annotation. One such example is the detection of all members of G-protein coupled receptor superfamily which are very diverse in nature often lacking significant sequence similarity. The high abundance of the members of this superfamily in human proteome (proteins with five functional annotations, Table 6) is in accordance with the results obtained using a hidden Markov model specially designed for this superfamily [42].

We have addressed the origin of functional domains in the entire human proteome by tracing them hierarchically along the *Homo sapiens *lineage. For better understanding of the evolutionary origin of these domains, we used a subtractive searching method, where the origin of a human domain is detected by hierarchically searching for its remote homologs in a database specific to an evolutionary node (see Methods). A common idea is that similar sequences should be searched with 'hard' matrices (created from less divergent sequences) and remote sequences should be searched with 'soft' matrices (built from more divergent sequences) [50]. When a human domain is searched against the entire dataset (containing sequences from all nodes) using PSI-BLAST, the PSSMs become too 'soft' meaning that they are better at finding remote homologs but not so in finding closer homologs. Since the domain repertoire in the human proteome is an incremental collection along its evolutionary lineage, soft matrices need not always be effective in finding all homologs. For this reason, the subtractive searching method adopted in this report creates both soft as well as hard matrices as appropriate depending on the node to be searched. For instance, to search bacterial and archael databases, PSSMs are created against eukaryota node (creates soft matrices) while, to search the mammalia node, PSSMs are created against primates node (creates hard matrices) and searched against 'other_mammalia' node that includes all mammalian sequences except those from primates. The unique combination of steps explained above has enabled us to efficiently detect human domains and to search for their origins along the lineage of *Homo sapiens*. It is indeed the differences in the sensitivity of HHpred and PSI_BLAST methods that help delineate the degree of divergence of functional domain families during the course of evolution (Figure 5). How far different functional families can diverge during evolution can be best represented by their power-law behavior (Figure 5A). Previous works [7,8] have reported about the scale-free network behavior of domain combinations revealing the fact that very few domain superfamilies can be connected with many different domains, while most of them remain adjacent to only one or two types of neighbors. Based on the functional importance of the highly connected domains within different proteomes, increase in complexity of multi-cellular from single-celled organisms was explained analogously [7]. In our study, we explain this gradation of functional importance or evolution of functional families in a quantitative way using the number of nodes associated with it. It is very interesting to note that functions which are related with fundamental processes of life and those very specific to a particular species are predominantly associated with a single node of origin (Table 4). In contrast, functions which have diverse applications in function space, such as cell-cell interactions, eukaryotic kinases, structure stabilizing factors, transmembrane receptors, etc., exhibit different degrees of divergence as necessitated by the complexity of proteomes. Thus, like domain combinations, domain evolution is also showing power-law behavior, which means few functional families undergo high degree of divergent evolution whereas most of the functional families generally evolve maintaining sequence identity detectable within the range of profile-sequence comparison method.

While domain evolution is best represented by its power-law behavior, protein evolution follows the exponential decay (Figure 8) along different nodes in *Homo sapiens *lineage. Thus, it is very unlikely for a protein sequence to undergo changes at every stage of evolution. In this study, we have seen a spectrum of patterns of protein sequences originated at different nodes of origin (Figure 9). We have observed a large number of Pfam-B assignments in proteins originated from multiple nodes, mainly at eukaryota, metazoa or chordata nodes (Figure 4). Despite the lack of definite functional annotation for these Pfam-B families, their conservation in multiple species (we used those families with at least 5 members) have implications in future research. Functional domains seen at early stages of evolution have evolved in various ways to cope with the complexity in multi-cellular organism while new functionalities specific to a node generally appear anew, not in combination with other already known domains.

## Conclusion

In conclusion, this work provides enhanced understanding of the origin of the human domain repertoire along its evolutionary lineage with implications in domain evolution as well as protein evolution of human proteins. The knowledge on the nodes of origin, frequency and combination of functional modules in proteins, functional versatility and the degree of divergence of these modules will provide better understanding of the evolutionary solution to the increased complexity of human proteome.

## Methods

### Selection of evolutionary nodes along the *Homo sapiens *lineage

The full evolutionary lineage for *Homo sapiens *was sub-divided into major hierarchical 'nodes' that include cellular organisms, eukaryota, metazoa, chordata, mammalia, primates and *Homo sapiens *(taxonomy ids are 131567, 2759, 33208, 7711, 40674, 9443 and 9606 respectively), where each 'node' represents a distinct group of species in the hierarchy, arranged in the ascending order (lower node to higher node) along the lineage (Figure 1). The term 'other_node' was used to denote a group of organisms excluding the next higher node in the lineage. For example, if node B is next to node A in the lineage, 'other_nodeA' refers to all species from 'nodeA minus nodeB'. Thus, for node eukaryota, 'other_eukaryota' refers to all species from eukaryota minus metazoan, and for node metazoa, 'other_metazoa' refers to all species under metazoa except those under chordata, and so on and so forth for other major nodes in the lineage (Figure 1). For cellular organisms, such 'other_node' consists of two different kingdoms of life, archaea and bacteria (with taxonomy ids, 2157 and 2, respectively). Hence they were treated as two separate subnodes under 'other_node' category and referred with their names.

### Data collection and preparation

We have collected the complete proteome of *Homo sapiens *containing 35,641 sequences from the Integr8 database (release 26) [51]. Protein sequences belonging to different nodes in the *Homo sapiens *lineage were retrieved from the NCBI taxonomy database [52,53] through E-Utilities program [collected by September 12th, 2005]. Datasets were cleaned by filtering out those annotated as 'environmental', 'unidentified', 'uncultured' and 'unclassified', and organized into datasets of nodes and other_nodes as explained above. For instance, sequences in other_eukaryota were collected by excluding all metazoan sequences from the eukaryota node. Sequences at each node (eukaryota, metazoa, chordata, mammalia, primates and *Homo sapiens*) and also sequences from archaea and bacteria were clustered at 90% sequence identity using CD-HIT program [54] to eliminate highly homologous sequences within each dataset. Number of protein sequences (after clustering) for archaea, bacteria and eukaryotic nodes are: for archaea 57,811; bacteria 9,14,421; eukaryota 7,62,692; metazoa 4,14,798; chordata 2,78,611; mammalia 1,80,488; primates 80,339 and for *Homo sapiens *61,939. Sequences in other_node databases (other_eukaryota, other_metazoa, other_chordata, other_mammalia and other_primates) were not clustered since they were derived from the clustered sets.

### Assignment of functional domains to the human proteome

We used domain definitions from the Pfam database version 19.0 [20] which is a collection of protein families compiled based on profile hidden Markov models (HMMs). This release contains 8,183 Pfam-A families. To identify functional domains in protein sequences, we used a sensitive HMM-HMM comparison method, HHpred [30,55], which employs both sequence and secondary structure information to identify remote homologs. HHpred builds a HMM for each query sequence following a series of steps that include: (i) searching the query sequence against the 'nr' (non-redundant) database using PSI-BLAST program [56]; (ii) multiple alignment of sequences obtained from the PSI-BLAST output; (iii) addition of secondary structure information to the multiple alignment, as predicted by PSIPRED program [57], and (iv) building a HMM for this multiple alignment. For PSI-BLAST searches, we have clustered the NCBI's non-redundant (nr) database to increase the speed of searching; clustering was done at 90% identity (nr90) for the first two iterations as PSI-BLAST requires more seed sequences to build up its position specific scoring matrices, followed by nr70 (clustered at 70% identity) for the next 2 iterations when searching for remote homologs with less sequence identity becomes more important. An inclusion E-value threshold of 1E-05 was used in both the steps. Each query HMM was searched against the Pfam-A database of HMMs (functionally known protein families) using HHSearch program version 1.2.0 (supplied in HHpred), to identify functional domains at a domain E-value cut-off of 1E-05, a very stringent cut-off with the probability of finding remote homolog as 95%. Protein sequences which are very small (less than 10 residues) and very large (greater than 6000 residues in length) cannot be detected by this method and hence they were excluded from our dataset. This functional domain detection step took ~ 60 hours for every 1000 query sequences using Dell's dual Xeon node processor with 3.2 GHz clock speed and 4 GB of RAM. We have used a compute-node cluster containing 16 dual node processors to carry out these large-scale computational tasks.

### Detection of conserved domains with unknown function

Segments of protein sequences with Pfam-A assignments were separated, and the rest of the proteome including full or partial sequences longer than 50 residues (unannotated human proteome) were searched against the Pfam-B database, version 19.0 to identify the conserved domains. Pfam-B families include conserved domains of unknown function that do not overlap with Pfam-A families and are automatically generated from the ProDom database [21]. We have selected only those Pfam-B families (30,463 families) with at least five members and built HMMs using HMMER 2.3.2 program. Conserved domains in the unannotated human proteome were identified by searching against Pfam-B HMMs at a domain E-value cut-off of 1E-05, using a faster 'hmmpfam' program supplied in the SledgeHMMER package [58].

### Pfam clans

Pfam-C database [33] version 19.0 was used to track the superfamily members (clans) classified in separate Pfam-A families. Pfam-C contains 205 clans where each clan contains more than one Pfam-A member families.

### Tracing the nodes of origin for human domains by subtractive searching method

In this method, we create target databases by 'subtracting' higher node sequences from a lower node database and then we 'search' human domains against them for detecting remote homologs – so we use the term 'subtractive searching' method for this type of tracing process. Assigned domains in the human proteome were traced for their first appearance (origin) along the *Homo sapiens *lineage (Figure 1), as shown in the flow diagram in Figure 2. We created PSSMs (Position Specific Scoring Matrices) for each human protein domain against a node database (source database), using 3 iterations of PSI-BLAST with an inclusion E-value threshold of 1E-05. These PSSMs were then used as scoring matrices to search against the lower other_node database (target database), using a single iteration of PSI-BLAST at an expectation value of 1E-05. For each human domain, remote homologs with an E-value lower than 1E-05 were retrieved from the target database. For those human domains with no hits at the target database, the same process is iterated against the next higher node followed by searching against lower other_node database. Hence, domains with remote homologs are subtracted at each node and only domains with no hits at the lower other_node databases will be forwarded to search against the next higher other_node until all other_nodes are searched along the *Homo sapiens *lineage. When a remote homolog of the domain is found at one other_node, then its node of origin will be its immediate lower node. That means, if a domain is found at other_mammalia node, then it's node of origin will be mammalia node. For detecting the origins of human protein domains in archaea or bacteria, we created PSSMs against the eukaryota node to search against archaeal and bacterial databases. Between archaea and bacteria, since it is difficult to say which one has earlier origin, we used three types of subnodes of origin depending on the occurrence of remote homologs, i.e. 'archaea_only' (remote homologs first found only in archaea, but not in bacteria), 'bacteria_only' (remote homologs first found only in bacteria but not in archaea) and 'archaea+bacteria' (remote homologs found both in archaea and bacteria). When a domain fails to find a homolog at none of the other_node databases, then it's node of origin will be considered as *Homo sapiens *which is the last node. This step of tracing the evolutionary origin took ~ 20 hours for every 1000 identified domains, using Dell's dual Xeon node processor with 3.2 GHz clock speed and 4 GB of RAM.

### Average percentage sequence identity within Pfam-A family members

We have used a pair-wise global alignment program, Needle (from EMBOSS version 3.0.0) [59] for determining percentage sequence identity among the human domains assigned to the same Pfam-A family with a gap opening penalty of 10.0 and gap extension penalty of 0.5. For a given Pfam-A family, average percentage sequence identity was calculated by taking the average of all-to-all pair-wise percentage identities between its members. Nevertheless for statistical reasons, Pfam-A families containing less than 10 members were eliminated and redundancy within families was removed by clustering at 100% identity.

## Authors' contributions

LRP carried out this work, analyzed the results and drafted the manuscript while, CG conceived of the study, designed the methodology, provided overall conceptual framework for this paper and contributed to manuscript preparation. All authors have read and approved the final manuscript.
